# Does overweight before pregnancy reduce the occurrence of gastroschisis?: the Japan Environment and Children’s Study

**DOI:** 10.1186/s13104-020-4915-7

**Published:** 2020-01-30

**Authors:** Takehiro Michikawa, Shin Yamazaki, Eiko Suda, Tatsuo Kuroda, Shoji F. Nakayama, Tomohiko Isobe, Yayoi Kobayashi, Miyuki Iwai-Shimada, Makiko Sekiyama, Toshihiro Kawamoto, Hiroshi Nitta, The Japan Environment and Children’s Study Group

**Affiliations:** 1 Department of Environmental and Occupational Health, School of Medicine, Toho University, 5-21-16 Omori-nishi, Ota-ku, Tokyo, 143-8540 Japan; 2 Centre for Health and Environmental Risk Research, National Institute for Environmental Studies, 16-2 Onogawa, Tsukuba, Ibaraki 305-8506 Japan; 3 Department of Paediatric Surgery, Keio University School of Medicine, 35 Shinanomachi, Shinjuku-ku, Tokyo, 160-8582 Japan

**Keywords:** Pre-pregnancy body mass index, Overweight, Gastroschisis, Birth cohort, Japan

## Abstract

**Objective:**

For several observational studies that have reported the factors related to gastroschisis, the target population in these studies was mainly residents of Europe or the US, and there is little data on the Asian population. In this study, we summarised characteristics of Japanese women who delivered infants with gastroschisis, particularly focusing on the pre-pregnancy body mass index (BMI), which was found to be inversely associated with gastroschisis in past studies, because the distribution of BMI is clearly different in Asia and the West.

**Results:**

We used data from a nationwide birth cohort study which recruited pregnant women between 2011 and 2014. Among 92,796 women who delivered singleton live births, the frequency of underweight (pre-pregnancy BMI < 18.5 kg/m^2^) was 16.2%, reference weight (18.5–24.9 kg/m^2^) 73.1%, and overweight (≥ 25.0 kg/m^2^) 10.6%. We identified only 9 infants with gastroschisis, 2 of whose women were underweight (frequency of gastroschisis = 0.01%), 5 were in the reference group (0.01%), and 2 were overweight (0.02%). Of these 9 women, none were aged < 20 years, 2 were aged 20–29 years (frequency = 0.01%), and 7 were aged 30–39 years (0.01%). No reduction in the occurrence of gastroschisis was apparent among Japanese women who were overweight before pregnancy.

## Introduction

Gastroschisis is a congenital abdominal wall birth defect, usually occurring to the right of the umbilicus [1]. Its observed frequency has differed by country and race: e.g., 2.5 per 10,000 births in Europe [2], and around 1 per 10,000 births in Japan [3, 4]. The majority of gastroschisis cases are isolated cases without associated anomalies; thus, infants with gastroschisis generally have a good vital prognosis [1]. On the other hand, gastroschisis is the leading cause of neonatal prolonged hospitalisation and expensive hospital costs [5]. Also, the childhood and adolescent health status of those who have experienced gastroschisis seems likely to be different from that of the general population. For example, many of those born with gastroschisis report abdominal pain at least once a week [6]; and compared with the general population, gastroschisis survivors tend to display a reduced working memory index, and behavioural and/or parental relationship impairment [7].

Though the pathogenesis of gastroschisis remains unclear, factors associated with the defect have been investigated through observational studies. However, the target populations in these studies were mainly residents of Europe or the US, and there is little data on the Asian population. Moreover, background characteristics differ between Asians and Westerners. Although a high frequency of gastroschisis has been observed among women in their teens [1], the percentage of Japanese teenage mothers is low (1.3% of women who gave births in the 2013 Vital Statistic Survey) [8]. Several studies have reported that an increase in pre-pregnancy body mass index (BMI) was associated with a decreased risk of gastroschisis [9–11]. However, the frequency of overweight and obesity is markedly lower in Asia than in the West [12, 13]. Therefore, the characteristics of Asian women who give birth to infants with gastroschisis do not necessarily correspond to those of similar non-Asian women.

The aim of this study was to summarise characteristics of Japanese women who delivered infants with gastroschisis, with particular focus on the frequency of gastroschisis based on the strata of pre-pregnancy BMI, which is distributed to the lower side in Japan.

## Main text

### Methods

#### Study participants

We used data from an ongoing nationwide birth cohort study, the Japan Environment and Children’s Study (JECS) [14, 15]. The JECS concept and design are described in detail elsewhere [16]. In brief, we recruited women as early in pregnancy as possible, in 15 Regional Centres throughout Japan, and registered 103,099 pregnancies between 2011 and 2014. After the exclusion of 2321 women who had no subsequent delivery record, the remaining 100,778 pregnancies involved 101,779 foetuses, and resulted in 100,148 live births. The selected characteristics of the women and children did not essentially differ between the JECS and general Japanese population [17]. The JECS protocol was approved by the Japan Ministry of the Environment’s Institutional Review Board on Epidemiological Studies, and the Ethics Committees of all participating institutions. All participants had provided written informed consent.

In this study, we restricted the study participants to 95,170 unique women (first JECS pregnancy), among the total of 100,778 pregnancies, which included women registered multiple times for sibling births. Of the 95,170 pregnancies, we excluded 947 twin or triplet pregnancies, and 1427 miscarriages or stillbirths. As a result, a total of 92,796 women who delivered singleton live births were included in our analysis.

#### Questionnaires and medical record transcription

We collected self-reported information, such as demographic, lifestyle, and physical and mental health factors, through questionnaires, and clinical information from medical record transcriptions. One self-administered questionnaire was distributed to the women during their first trimester, and another during their second or third trimester. Medical record transcriptions adhering to the JECS in-house standard operating procedures were performed three times by physicians, midwives or nurses, and/or Research Co-ordinators: first during the first trimester, second after delivery, and finally at the first-month health check-up after delivery.

The pre-pregnancy maternal height and weight, obtained via the medical record, were used to calculate the pre-pregnancy BMI as weight (kg)/height squared (m^2^). As we included a question about pre-pregnancy height and weight in the first questionnaire, such questionnaire-based information was used as a fallback measure. The Pearson’s correlation coefficients between the medical record and questionnaire were 0.99 for height and 0.98 for weight before pregnancy. Based on the WHO criteria [18], the pre-pregnancy BMI was categorised into three groups: < 18.5 (underweight), 18.5–24.9 (reference), ≥ 25.0 kg/m^2^ (overweight). Other characteristics we summarised were maternal age at delivery (< 20, 20–29, 30–39, ≥ 40 years), occupation in early pregnancy (administrative, managerial, professional, or engineering; clerical; sales and service; homemaker; others), smoking habits (never smoked, ex-smoker or smoker during early pregnancy), alcohol consumption (never drank, ex-drinker or drinker during early pregnancy), current history of diabetes or gestational diabetes (no, yes), parity (0, ≥ 1), infertility treatment (no, yes), routine use of folic acid supplement (no, yes (≥ 4 times/week)), week of pregnancy at delivery (< 37, ≥ 37), and infant sex.

The transcription reports after delivery and at a month after delivery contained a list of congenital anomalies, including gastroschisis. When gastroschisis was indicated in either report, we considered it to indicate a case of gastroschisis in this study.

#### Statistical analysis

The baseline characteristics of the women were summarised. The association between pre-pregnancy BMI and gastroschisis was investigated using a logistic regression model with penalisation approach to minimise sparse data bias [19], and the maternal age adjusted odds ratios (ORs) and 95% confidence intervals (CIs) of gastroschisis were estimated. In one case mother (age category: 30–39 years), data on the mother’s pre-pregnancy height and weight was missing; however, we had information on her weight just before delivery (57.5 kg), and using the average weight gain during pregnancy in this population (10.3 kg), we estimated her pre-pregnancy weigh to be 47.2 kg. Further, according to the 2014 Japanese National Health and Nutritional Survey [20], the mean height in women aged 30–39 years old was 158 cm; therefore, we estimated her pre-pregnancy BMI to be 18.9 kg/m^2^.

This study used the dataset jecs-ag-20160424, which was released in June 2016, and revised in October 2016, along with the supplementary dataset jecs-ag-20160424-sp1. All analyses were performed by using Stata 14 (StataCorp LP, College Station, Texas, USA).

### Results

The baseline characteristics of the 92,796 women (mean age at delivery = 31.2 years, standard deviation (SD) = 5.1) are presented in Table 1. With respect to the pre-pregnancy BMI (mean = 21.2 kg/m^2^, SD = 3.3), 16.2% were categorised as underweight, 73.1% as reference, and 10.6% as overweight. The percentage of obesity (a BMI of ≥ 30 kg/m^2^) was 2.5%. In this population, we identified 9 infants with gastroschisis (1.0/10,000 live births). Among these 9, 2 were born from underweight women (frequency of gastroschisis = 0.01%), 4 from those in the reference group (0.01%), 2 from overweight women (0.02%), and in one case data on the mother’s pre-pregnancy height and weight was lacking. Women aged < 20 years accounted for 0.9%, those aged 20–29 years 36.6%, those aged 30–39 years 57.9%, and those aged ≥ 40 years 4.6%, of the total number of women. Two women aged 20–29 years delivered infants with gastroschisis (frequency of gastroschisis = 0.01%), and 7 women aged 30–39 years (0.01%). There were no cases in the < 20 or ≥ 40 groups. Also, no cases were observed in the current history of diabetes or gestational diabetes group, or in the infertility treatment group. The frequency of gastroschisis tended to be higher in the preterm (< 37 weeks of pregnancy) birth group. No noteworthy difference based on the strata of other characteristics, such as smoking status, alcohol consumption, or parity, was found.

**Table 1:** Baseline characteristics of 92,796 women who delivered singleton live births

	Number of women	Frequency (%)	Gastroschisis (n = 9)	
			Number of cases	Frequency (%)
Pre-pregnancy body mass index				
< 18.5 kg/m^2^ (underweight)	15,034	16.2	2	0.01
18.5–24.9 kg/m^2^ (reference)	67,774	73.1	4	0.01
≥ 25.0 kg/m^2^ (overweight)	9864	10.6	2	0.02
Missing	124	0.1	1	
Age at delivery (years)				
< 20	854	0.9	0	0
20–29	33,955	36.6	2	0.01
30–39	53,683	57.9	7	0.01
≥ 40	4299	4.6	0	0
Missing	5	0.0	0	
Occupation in early pregnancy				
Administrative, managerial, professional, or engineering	20,988	22.6	0	0
Clerical	15,501	16.7	3	0.02
Sales and service	20,118	21.7	3	0.01
Homemaker	25,193	27.2	2	0.01
Others	8965	9.7	0	0
Missing	2031	2.2	1	
Smoking habits				
Never smoked	53,336	57.5	3	0.01
Ex-smoker or smoker during early pregnancy	38,486	41.5	6	0.02
Missing	974	1.1	0	
Alcohol consumption				
Never drank	31,681	34.1	5	0.02
Ex-drinker or drinker during early pregnancy	60,231	64.9	4	0.01
Missing	884	1.0	0	
Current history of diabetes or gestational diabetes				
No	89,871	96.9	9	0.01
Yes	2925	3.2	0	0
Parity				
0	40,420	43.6	3	0.01
≥ 1	51,968	56.0	5	0.01
Missing	408	0.4	1	
Infertility treatment				
No	86,532	93.3	8	0.01
Yes	6140	6.6	0	0
Missing	124	0.1	1	
Routine use of folic acid supplement				
No (< 4 times/week)	66,172	71.3	5	0.01
Yes (≥ 4 times/week)	24,548	26.5	3	0.01
Missing	2076	2.2	1	
Week of pregnancy at delivery				
< 37	4364	4.7	6	0.14
≥ 37	88,210	95.1	3	0.00
Missing	222	0.2	0	
Infant sex				
Male	47,603	51.3	7	0.01
Female	45,182	48.7	2	0.00
Unknown or missing	11	0.0	0	

The association between pre-pregnancy BMI and gastroschisis is summarised in Table 2. This includes the aforementioned mother, for whom there was no pre-pregnancy data for height and weight, with a postulated BMI of 18.9 kg/m^2^. When she was categorised into either the < 18.5 kg/m^2^ or the 18.5–24.9 kg/m^2^ group, the OR point estimates for the overweight group were above unity, in comparison with the reference (see Additional file 1). In addition, we re-categorised two women in the underweight group as members of the reference group, because their BMI values were near 18.5 kg/m^2^. Nonetheless, the resulting point estimates showed no direction of decreased risk of gastroschisis in the overweight group (OR = 2.0, 95% CI 0.4–9.8). In the case of two women in the overweight group (one in her 20 s and another one in her 30 s), since their BMI values were 28, it was unlikely that BMI category misclassification occurred.

**Table 2:** Odds ratios (ORs) of gastroschisis based on the pre-pregnancy body mass index (BMI)

Pre-pregnancy BMI	No. of women	No. of gastroschisis cases	Frequency of gastroschisis	Maternal age at delivery adjusted OR	95% confidence interval
Complete case analysis					
< 18.5 kg/m^2^ (underweight)	15,034	2	0.01	2.2	(0.4–11.0)
18.5–24.9 kg/m^2^ (reference)	67,774	4	0.01	Reference	
≥ 25.0 kg/m^2^ (overweight)	9864	2	0.02	3.4	(0.6–17.8)
(Situation 1) we categorised one mother^a^, who delivered an infant with gastroschisis and had no information on height and weight before pregnancy, as in the reference group					
< 18.5 kg/m^2^ (underweight)	15,034	2	0.01	1.7	(0.4–8.3)
18.5–24.9 kg/m^2^ (reference)	67,775	5	0.01	Reference	
≥ 25.0 kg/m^2^ (overweight)	9864	2	0.02	2.7	(0.5–13.5)
(Situation 2) we categorised one mother^a^, who delivered an infant with gastroschisis and had no information on height and weight before pregnancy, as in the underweight group.					
< 18.5 kg/m^2^ (underweight)	15,035	3	0.01	3.1	(0.7–13.6)
18.5–24.9 kg/m^2^ (reference)	67,774	4	0.01	Reference	
≥ 25.0 kg/m^2^ (overweight)	9864	2	0.02	3.5	(0.7–18.2)
(Situation 3) given situation 1, we re-categorised two cases of women with BMI < 18.5 kg/m^2^ as women of the reference group, because their BMI values were near 18.5 kg/m^2^					
< 18.5 kg/m^2^ (underweight)	15,032	0	0		
18.5–24.9 kg/m^2^ (reference)	67,777	7	0.01	Reference	
≥ 25.0 kg/m^2^ (overweight)	9864	2	0.02	2.0	(0.4–9.8)

^a^ We had information on her weight just before delivery (57.5 kg), and using the average weight gain during pregnancy in this population (10.3 kg), we estimated her pre-pregnancy weigh to be 47.2 kg. Further, according to the 2014 Japanese National Health and Nutritional Survey, the mean height in women aged 30–39 years old was 158 cm; therefore, we estimated her pre-pregnancy BMI to be 18.9 kg/m^2^

### Discussion

In this study, overweight women did not show a reduced likelihood of giving birth to infants with gastroschisis. This result would appear to contradict those of a majority of other related studies, which reported that a high BMI before pregnancy was protectively associated with gastroschisis [9–11]. However, the result does not strike us as surprising. First, a markedly low risk of gastroschisis has been observed among obese women (a BMI of ≥ 30.0 kg/m^2^) [9, 21, 22]. The prevalence of obesity among women aged 20–49 years was 28.7% in the US National Health and Nutritional Examination Survey of 2007–2010, and 22.1% in the Health Survey for England 2008–2009 [23], whereas the prevalence in this study population was only 2.5%. We found no cases of gastroschisis births among obese women. Also, it was understandable that women with diabetes or gestational diabetes, which involve the same metabolic disorders as obesity [24], had no infants with gastroschisis.

A high frequency of gastroschisis births has been observed among teenage women [1]. One case–control study in UK reported that the link between low BMI and gastroschisis was explained by younger mothers being thinner [25]. Teenage childbirth is not uncommon internationally; for instance, 7.0% of US women were under 20 years of age in 2013 [26]. However, the percentage is very low in Japan (~ 1%) [8], roughly reflecting the result in this study (0.9%). Infants with gastroschisis were mainly born from women aged 30–39 years, who accounted for roughly 60% in this cohort. Therefore, younger women particularly did not deliver infants with gastroschisis. Certainly in Japan, age is weakly but positively correlated with BMI [20]. Unlike other non-Asian population, young maternal age and lower BMI seem not to be associated with the occurrence of gastroschisis in Japanese population.

As in past studies (e.g., [1]), the frequency of gastroschisis births was high in the preterm delivery group. Although maternal smoking and alcohol consumption may be risk factors for gastroschisis [27], we observed no clear difference in gastroschisis frequency based on smoking or alcohol consumption status. In Japan, the BMI distribution tends toward lower values, the average maternal age at delivery is above 30 years old [8], and people have distinctive lifestyle (e.g., dietary habits), in comparison with the West. Gathering epidemiological evidence regarding the risk profile of gastroschisis in Asia may play a part in the elucidating less well-understood aspects of the aetiology of gastroschisis.

We found that no decrease in the occurrence of gastroschisis was apparent among Japanese women who were overweight before pregnancy.

## Limitations

We identified a small number of cases of gastroschisis (n = 9), although this was a large-scale birth cohort, and the frequency of gastroschisis here observed (1.0/10,000 live births) was within the range of past reports in Japan [3, 4]. Therefore, the low number of cases limited data analysis and conclusions being drawn; that is, we could not summarise certain characteristics, such as maternal medication, especially opioid use [28], and could not statistically discuss the differences in frequency of gastroschisis based on the strata of individual characteristics (for example, only 4 cases in the reference BMI group). Nonetheless, we considered that the descriptive data on Japanese gastroschisis frequency in this context was worthy of report, to encourage and contribute to future studies, such as case–control design considered statistical justification of sample size, regarding gastroschisis-related factors among Asians, who have the lower percentage of overweight and obesity compared with Caucasians.

## Supplementary information

**Additional file 1:** Association between pre-pregnancy body mass index and gastroschisis. Odds ratios were adjusted for maternal age at delivery. Error bars indicate 95% confidence intervals.

## Data Availability

Data are unsuitable for public deposition due to ethical restrictions and legal framework of Japan. It is prohibited by the Act on the Protection of Personal Information (Act No. 57 of 30 May 2003, amendment on 9 September 2015) to publicly deposit the data containing personal information. Ethical Guidelines for Medical and Health Research Involving Human Subjects enforced by the Japan Ministry of Education, Culture, Sports, Science and Technology and the Ministry of Health, Labour and Welfare also restricts the open sharing of the epidemiologic data. All inquiries about access to data should be sent to: jecs-en@nies.go.jp. The person responsible for handling enquiries sent to this e-mail address is Dr Shoji F. Nakayama, JECS Programme Office, National Institute for Environmental Studies.

## Abbreviations

BMI: Body mass index
CI: Confidence interval
JECS: Japan Environment and Children’s Study
OR: Odds ratio
