# Supplementary material for: Does overweight before pregnancy reduce the occurrence of gastroschisis?: the Japan Environment and Children’s Study
Source: BMC Res Notes. 2020 Jan 30;13:47. doi: 10.1186/s13104-020-4915-7 (PMC6990474; doi:10.1186/s13104-020-4915-7)
Supplement: Supplementary file 1 — Additional file 1: Association between pre-pregnancy body mass index and gastroschisis. Odds ratios were adjusted for maternal age at delivery. Error bars indicate 95% confidence intervals. [file 13104_2020_4915_MOESM1_ESM.pdf]

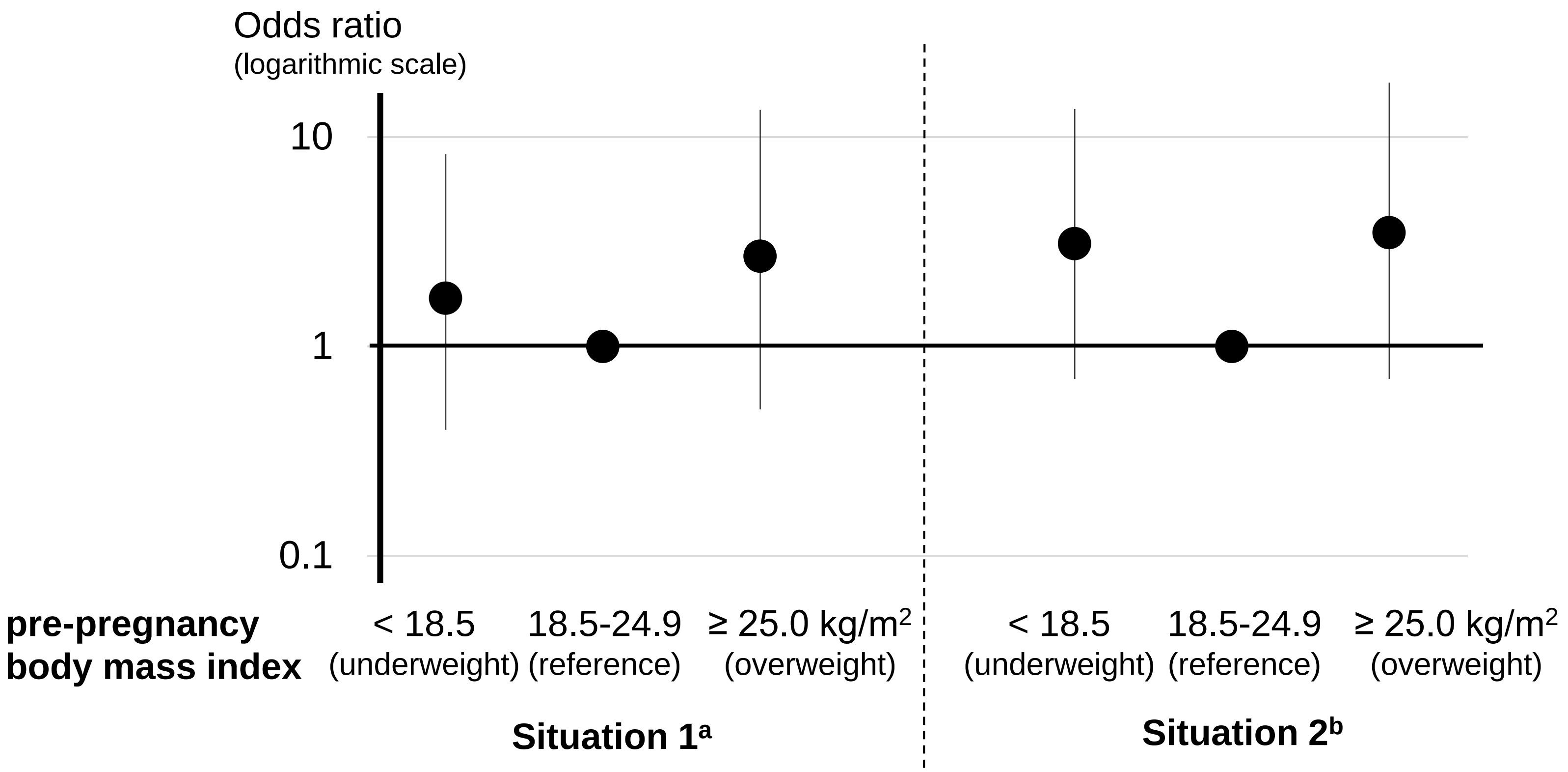

Additional file 1

Association between pre-pregnancy body mass index and gastroschisis.

Odds ratios were adjusted for maternal age at delivery. Error bars indicate 95% confidence intervals.

[a] We categorised one mother[c], who delivered an infant with gastroschisis and had no information on height and weight before pregnancy, as in the reference group.

[b] We categorised one mother[c], who delivered an infant with gastroschisis and had no information on height and weight before pregnancy, as in the underweight group.

[c] We had information on her weight just before delivery (57.5 kg), and using the average weight gain during pregnancy in this population (10.3 kg), we estimated her pre-pregnancy weigh to be 47.2 kg. Further, according to the 2014 Japanese National Health and Nutritional Survey, the mean height in women aged 30-39 years old was 158 cm; therefore, we estimated her pre-pregnancy body mass index to be 18.9 kg/m^2.
